# Further Evidence of Early-Onset Osteoporosis and Bone Fractures as a New *FGFR2*-Related Phenotype

**DOI:** 10.3390/ijms26094204

**Published:** 2025-04-29

**Authors:** Alice Moroni, Elena Pedrini, Morena Tremosini, Alessia Di Cecco, Dario Cocciadiferro, Antonio Novelli, Lucia Santoro, Rosanna Cordiali, Luca Sangiorgi, Maria Gnoli

**Affiliations:** 1 Department of Rare Skeletal Disorders, IRCCS Istituto Ortopedico Rizzoli, 40136 Bologna, Italyalessia.dicecco@ior.it (A.D.C.); maria.gnoli@ior.it (M.G.); 2 Translational Cytogenomics Research Unit, Bambino Gesù Children’s Hospital, IRCCS, 00165 Rome, Italy; 3 Department of Clinical Sciences, Division of Pediatrics, Azienda Ospedaliero Universitaria delle Marche, Presidio Salesi, 60126 Ancona, Italy

**Keywords:** osteoporosis, *FGFR2*, FGF signaling, early-onset osteoporosis, osteogenesis, osteogenesis imperfecta

## Abstract

Primary osteoporosis in children and young adults often suggests a monogenic disease affecting bone microarchitecture and bone mineral density. While Osteogenesis Imperfecta (OI) is the most recognized genetic cause of recurrent fractures, many other genes involved in bone metabolism may contribute to osteoporosis. Among them, FGFR2 plays a critical role in bone growth and development by regulating osteoblast differentiation and proliferation, as well as chondrogenesis. Germline pathogenic *FGFR2* variants are typically associated with syndromic craniosynostosis, conditions not characterized by bone fragility or osteoporosis. A report recently identified FGFR2 as a potential cause of dominant early-onset osteoporosis and bone fractures in a family. We report the case of a child affected by severe osteoporosis with multiple fractures. We performed clinical exome sequencing in trio to investigate potential genetic causes of the observed phenotype and identified a likely mosaic pathogenic *FGFR2* variant, absent in both parental samples. Our findings provide further evidence that *FGFR2* pathogenic variants can lead to a novel non-syndromic bone mineralization disorder, reinforcing the role of FGFR2 in the pathogenesis of early-onset osteoporosis.

## 1. Introduction

Osteoporosis is characterized by reduced bone density and abnormal bone microarchitecture, leading to bone fragility and increased risk of fractures [1]. It is a complex disorder and can be both a secondary symptom of other underlying conditions, with several risk factors having a role in its pathogenesis [2], and a primary disease where the bones become fragile and more prone to fractures due to a loss of mass and density. When occurring in childhood or young adulthood in the absence of an underlying causative condition, osteoporosis can be related to a monogenic bone disease [3]; in fact, bone fragility is a key finding in several skeletal dysplasias [4]. Several genes have been related to monogenic early-onset osteoporosis or juvenile osteoporosis [4,5]; the rapid advances in, and widespread availability of, molecular techniques - such as next-generation sequencing, exome sequencing, and whole-genome sequencing - have contributed to elucidating the molecular mechanisms underlying the disease.

As a paradigm of skeletal dysplasia with bone fragility, Osteogenesis Imperfecta (OI) represents a group of hereditary connective tissue disorders with recurrent fractures as main clinical manifestation [6]. *COL1A1* and *COL1A2* mutations account for about 85–90% of cases of OI; despite this, many other genes involved in collagen biosynthesis, bone mineralization, and osteoblast differentiation have been identified as causative of both OI and hereditary early-onset osteoporosis, thus expanding the molecular mechanisms of bone fragility [7,8,9]. In 2023, the revised *Nosology of genetic skeletal disorders* included over 50 genetic conditions in the “Osteogenesis imperfecta and bone fragility group” [4].

Despite advances in the understanding of genetic disorders, pathogenic variants in the known genes account for only a small percentage of early-onset osteoporosis cases. In fact, a large study reported that approximately 20 genes associated with Osteogenesis Imperfecta (OI) and early-onset osteoporosis explained less than 20% of early-onset osteoporosis cases, suggesting the existence of many yet unidentified genetic factors involved in bone fragility [10].

Recently, Dantsev and colleagues [11] reported the case of a 13-year-old boy affected by osteoporosis and multiple fractures, with a family history of abnormal bone mineralization and fractures, carrying a heterozygous variant in the *FGFR2* gene. The authors suggested that autosomal dominant early-onset osteoporosis with bone fragility may represent a new clinical phenotype related to *FGFR2* pathogenic variants [11].

Fibroblast growth factor receptor 2 (FGFR2) is a tyrosine kinase receptor belonging to a family that includes four high-affinity receptors with a similar structure [12]. It is expressed in various tissues, and regulates many biological processes such as cell proliferation, migration, survival, and differentiation [13,14,15], with a key role in the development and growth of the skeleton [16]. In particular, FGFR2 is involved in the osteogenesis of cranial and long bones [17,18]. Moreover, studies in mouse models have revealed a critical role of both gain and loss-of-function *FGFR2* variants in balancing the proliferation and differentiation of osteoprogenitor cells [19,20,21].

Pathogenetic variants in *FGFR2* have been associated with various phenotypes, with craniosynostosis as the main clinical finding in most of them. In fact, in the revised nosology, *FGFR2*-related conditions are classified as “Craniosynostosis disorders” or “Bent bone disorders”, but not under “Osteogenesis Imperfecta and bone fragility”, as osteoporosis and bone fractures are not major clinical findings in *FGFR2*-related diseases [4]. The main *FGFR2*-related phenotypes are summarized in Table 1 [5].

In this report, we describe the case of a patient affected by severe osteoporosis with multiple fractures and negative molecular tests in genes commonly associated with bone fragility. Through a broader exome analysis, we detected a likely mosaic pathogenic variant in *FGFR2*. These findings further support that isolated primary osteoporosis could be a new *FGFR2*-related phenotype, as suggested by Dantsev et al., thus expanding the spectrum of genes responsible for bone fragility [11].

## 2. Results

### 2.1. Clinical Description

The boy was the first child of healthy non-consanguineous parents. No other family members showed signs of Osteogenesis Imperfecta and no early-onset osteoporosis or skeletal dysplasia cases in the family have been noted.

Natural childbirth occurred at 37 weeks of gestation (weight: 3680 g; length: 52 cm; Apgar score: 9–10). The main milestones in early psychomotor development were at appropriate ages.

At the age of 5 years and 4 months, the child underwent investigations due to knee pain and gait anomalies, in the absence of any history of trauma or signs of infection. An X-ray showed reduced mineralization and metatarsal abnormalities. Idiopathic arthritis was initially suspected.

The first bone fracture (non-traumatic fracture of left distal tibial metaphysis) occurred at age 6. At 7 years old, the proband presented T3-T4-T5 vertebral compression fractures, with a reduction in all but one high thoracic vertebral body, and bilateral VII rib non-traumatic fractures. He presented lower limbs pain and used to walk with aids. Clinical presentation and personal history were suggestive of a bone mineralization disorder. X-rays performed in the chest, femur, dorsal column, and right hand revealed reduced bone density. Dual-energy X-ray absorptiometry (DEXA) showed a Z-score of −3 at the lumbar level and a total body Z-score of −1.6.

Glomerular and tubular renal functions were normal. Celiac disease markers, rheumatoid factor, and ANA were all negative. Metabolic tests including plasma acylcarnitine, urinary organic acids, amino acids, and urinary mucopolysaccharides were normal. No alterations were reported in calcium–phosphorus metabolism, in thyroid function, in ACTH and cortisol values, in the GH-IGF1 axis, in blood gas analysis or in muscle enzymes. In addition, the evaluation of biochemical markers for bone status assessment revealed normal osteocalcin but markedly altered values of C-terminal telopeptide (CTX) (1175 pg/mL; normal values < 580 pg/mL) and procollagen type 1 N-terminal propeptide (P1NP) (455 mcg/L; normal range 15–60 mcg/L). The other marker values were as follows: serum calcium 9.6 mg/dL (normal range 8.5–10.5 mg/dL), serum phosphate 5.6 mg/dL (normal range 3.7–5.6 mg/dL), PTH 15 pg/mL (normal range 14–85 pg/mL), urinary calcium 14.9 mg/dL (normal range 0.9–37.9 mg/dL), alkaline phosphatase 289 U/L (normal values < 435 U/L), bone alkaline phosphatase 77.6 mcg/L (normal range 4–21 mcg/L), 25-OH-D2+D3 32.1 ng/mL (normal values > 30 ng/mL).

Cardiac and abdominal ultrasound revealed no abnormalities, with the only exception being the accessory spleen. Considering the fracture history and the DEXA results, quarterly bisphosphonate infusions (Neridronate) were started.

Despite treatment, the child experienced other fractures: a femoral neck fracture from minor trauma (7 years), a diaphyseal stress fracture of the right tibia (7 years and 6 months), a diaphyseal fracture of the right radius (8 years), and a pathological right femur fracture that was surgically treated (8 years and 8 months). Due to the lack of clinical benefit, bisphosphonate therapy was suspended after approximately two years.

Currently, at 11 years old, the proband walks indoors with a walker and navigates outside with a wheelchair, practicing only a few steps on his own. Hydrotherapy is performed 1 day/week and physiotherapy 2 h/week. He visits the emergency room monthly for musculoskeletal pain that does not respond to paracetamol, NSAIDs (nonsteroidal anti-inflammatory drugs), magnesium, and pregabalin. As of the clinical examination, the boy weighs 45 kg (90° pct) and is 130 cm tall (25–50° pct), with a BMI of 26.6 (mild obesity). He presents shortened trunk due to vertebral deformity, dorso-lumbar hyper lordosis and dorsal kyphosis in reduction, a protruding abdomen, a valgus knee, tibia varus, pronated feet, soft skin, mild hypotonia, and mild joint hyperlaxity, especially at the hips, with a Beighton score of 4.

### 2.2. Molecular Findings

To better delineate the clinical presentation, genetic tests were performed with the parents’ written informed consent. DNA was extracted from a peripheral blood sample and analyzed by Next-Generation Sequencing (NGS) to identify potential pathogenic variants. The initial analysis focused on genes associated with Osteogenesis Imperfecta and Hypophosphatasia, which yielded negative results. Subsequently, the analysis was extended to include all known disease-associated genes.

The clinical exome analysis revealed the presence of the mosaic (~30%) variant c.1262G>A p.(Arg421His) in the *FGFR2* gene (NM_000141.5). The variant has an allele frequency of 0.000005576 in the GnomAD database (https://gnomad.broadinstitute.org/ (accessed on 23 April 2025)), it has never been reported in the scientific literature, and it is predicted by Mutation Taster and PolyPhen 2.0 to be deleterious and probably damaging, with a CADD score of 31; according to the ACMG criteria (PP2  +  PP3 + PS2), the variant is classified as likely pathogenic (class 4).

## 3. Discussion

Bone development and homeostasis are complex processes that rely on a delicate balance between bone formation and resorption, which is essential for maintaining skeletal integrity and preventing conditions such as osteoporosis [1]. Osteoporosis is a multifactorial disease resulting from the interaction of genetic factors, environmental influences, lifestyle, comorbidities, and other contributing elements. Previous studies have highlighted a substantial genetic contribution to the disease. In particular, in cases of ‘primary’ early-onset osteoporosis, a few rare genetic variants with large effect sizes appear to play a significant role in its pathogenesis [22]. Genetic alterations affecting components involved in this balance can lead to monogenic skeletal disorders characterized by bone fragility, with Osteogenesis Imperfecta representing the paradigm. However, not all patients presenting with an OI-like phenotype carry pathogenic variants in known OI-related genes or in other genes commonly associated with bone fragility [6]. Moreover, several atypical forms of OI have been described, and many other mineralization disorders have been included in the most recent nosology [4], complicating the clinical characterization and differential diagnosis of patients with recurrent fractures. Recent studies have further expanded our understanding of bone mineralization disorders by identifying novel causative genes involved not only in collagen biosynthesis and folding, but also in other molecular pathways that regulate bone development and homeostasis [6]. Despite these advances, a definitive molecular diagnosis can still be established in a small percentage of cases, leaving the pathogenesis of many forms of Osteogenesis Imperfecta and early-onset osteoporosis largely unexplained.

Herein we describe a patient with bone fragility and severe early-onset osteoporosis carrying a likely mosaic pathogenic variant in *FGFR2*. To our knowledge, this is the second report of early-onset osteoporosis in the presence of a *FGFR2* variant. In both our patient and a previously reported case [11], the clinical phenotype initially suggested a diagnosis of Osteogenesis Imperfecta; however, molecular investigations did not reveal any pathogenic variants in genes known to be associated with OI or bone fragility. In the previous report, the c.722dup *FGFR2* variant was identified in a young boy with recurrent fractures beginning from infancy, low BMD, joint pain, dental caries, and headaches. The same variant was also identified in the father, who had low-impact fractures, and in the sister, who presented with genu valgum, hip dysplasia, scoliosis, and dental caries, but had no fracture history [11].

FGFR2 is a tyrosine kinase receptor mainly expressed in the epithelial and mesenchymal cells, playing a key role in the development of endocrine glands, skeleton, skin, and other organs [23]. It is known to regulate the development of osteoblasts, and increased FGF/FGFR-dependent signaling can lead to hyperplasia of immature osteoblasts, inhibit their differentiation, and trigger apoptosis, causing an imbalance between osteosynthesis and bone resorption [13]. Recent findings also suggest that FGFR2 may contribute to chondrocytes development [14].

Pathogenic variants in *FGFR2* are primarily associated with skeletal dysplasias characterized by craniosynostosis as a key feature, while bone fragility and osteoporosis are not commonly recognized manifestations. Although the role of FGFR2 signaling in craniofacial skeletal development is well established [16,17], its function in the development of the appendicular skeleton remains less understood.

Notably, mineralization defects and long-bone deformities are indeed hallmarks of Bent Bone Dysplasia (OMIM #614592), a lethal *FGFR2*-related disease characterized by high perinatal lethality, bent long bones, osteopenia, craniosynostosis, and dysmorphic facial features. Studies suggest that impaired FGF signaling underlies these skeletal abnormalities, as indicated by reduced FGFR2 levels at the plasma membrane and decreased receptor responsiveness [24]. Similarly, mouse models carrying specific *FGFR2* splice variants exhibit delayed mineralization of the calvarium, craniosynostosis, and shortened long bones, further supporting the role of FGFR2 in bone formation [21].

Other in vitro and mouse model studies have demonstrated that FGFR2 is a regulator of bone construction, promoting osteoprogenitor cell proliferation and differentiation, and contributing to intramembranous bone formation and ossification processes [25]. These findings provide a plausible explanation for the mineralization defects observed in Bent Bone Dysplasia and align with the phenotype observed in our patient. In addition, FGFR2 directs the inhibition of WNT signaling in skull development [26]. Biallelic pathogenic *WNT1* (wingless-type mmtv integration site family, member 1) variants are associated with Osteogenesis Imperfecta, and monoallelic pathogenic variants cause an autosomal dominant form of early-onset osteoporosis. *WNT1* encodes a key protein in bone development and bone mass; therefore, we speculate that the involvement of FGFR2 in WNT signaling in bone tissue could lead to an osteoporosis phenotype.

Previous genome-wide association studies (GWASs) revealed the significant genetic contribution to the pathogenesis of osteoporosis, but common variants or loci cumulatively explain less than 20% of bone density variability [27,28,29]. Moreover, recent population studies have suggested an association between *FGFR2* variants and increased susceptibility to osteoporosis, further highlighting its contribution to bone homeostasis [30,31,32,33].

The molecular mechanism underlying the variable phenotypes associated with mutations in *FGFR2* is not completely understood, but a few genotype–phenotype correlations have been identified. Loss-of-function *FGFR2* mutations in the tyrosine kinase domain are responsible for lacrimo-auriculo-dento-digital syndrome (LADD, OMIM #123790), which is characterized by lacrimal duct aplasia, hearing loss, dental abnormalities, and digital malformations [34]. On the other hand, loss-of-function missense *FGFR2* variants in the trans-membrane (TM) domain have been found in Bent Bone Dysplasia [24]. The other syndromic *FGFR2*-related diseases are apparently caused by gain-of-function variants, leading to constitutive receptor activation or altered ligand specificity [16].

Our patient presents early-onset osteoporosis with recurrent fractures, joint pain, limb deformities, and impaired ambulation. He harbors a mosaic missense variant in *FGFR2*, probably related to the phenotype; however, in the absence of functional studies, we cannot exclude a different genetic or complex cause of the disease. The phenotype observed in our patient only partially overlaps with the Dantes and colleagues’ case, with fractures and osteoporosis as shared features (see Table 2). However, significant clinical variability was also reported among the previously described individuals, highlighting the variable clinical effects of these genetic alterations [11]. Furthermore, the variant we identified is a mosaic missense mutation, which may account for the phenotypic differences between the two reports. However, given the different location and type of variant, it is not possible to draw conclusions regarding a potential genotype–phenotype correlation. Our patient also differs in terms of their treatment response to bisphosphonates, suspended because they were not effective, whereas zoledronic acid appeared to be effective in the case reported by Dantsev et al. [11], which suggests that the treatment failure in our patient may be attributed to individual differences in drug sensitivity, a phenomenon already observed with bisphosphonates.

Further investigations are needed to elucidate the molecular mechanisms and pathways underling FGFR2 signaling and its role in various phenotypes, including bone mineralization defects. In relation to our case, assessing the level of mosaicism across different tissues, along with functional studies on bone tissue, may help clarify the molecular and biochemical alterations responsible for the patient’s phenotype.

Additional studies aimed at better characterizing bone density and architecture in more patients with *FGFR2*-related osteoporosis— for example, using high-resolution peripheral quantitative computed tomography—could help to define this new condition.

Clarifying the mechanism leading to *FGFR2*-related bone mineralization disorders and understanding its role in bone development and growth could also provide new insights to address new therapeutic approaches. Indeed, the identification of novel molecular mechanisms in OI has already paved the way for innovative treatment strategies [35].

## 4. Materials and Methods

After obtaining informed consent for the genetic analyses, clinical exome enrichment and parallel sequencing were performed on genomic DNA extracted from patients and parents’ circulating leukocytes. Library preparation and clinical exome capture were performed by using the Twist Custom Panel (clinical exome—Twist Bioscience) according to the manufacturer’s protocol and sequenced on the Illumina NovaSeq 6000 platform. The BaseSpace pipeline and Geneyx software 6.0 (LifeMap Sciences, Covina, CA, USA) were, respectively, used for variant calling and annotation. Sequencing data were aligned to the hg19 human reference genome. The functional impact of the variants was analyzed by Combined Annotation Dependent Depletion (CADD) V.1.3, Sorting Intolerant from Tolerant (SIFT 1.2), and Polymorphism Phenotyping v2 (PolyPhen-2). Rare variants (MAF < 0.1%) were filtered based on the gnomAD database. Based on the guidelines of the American College of Medical Genetics and Genomics, a minimum depth coverage of 30x was considered suitable for analysis. Variants were examined for coverage and Qscore (minimum threshold of 30) and visualized by the Integrative Genome Viewer (IGV).

## 5. Conclusions

Here, we describe the case of a child with severe osteoporosis and multiple fractures, carrying a likely mosaic pathogenic *FGFR2* variant. This report provides further evidence supporting osteoporosis as a new distinct phenotype associated with *FGFR2* variants. Our findings highlight the need for further investigations to unravel the role of *FGFR2* in bone development and growth. A deeper understanding of the molecular mechanism by which *FGFR2* contributes to osteoporosis could pave the way for identifying novel therapeutic targets for skeletal disorders characterized by bone fragility or multifactorial osteoporosis.

## Figures and Tables

**Table 1:** Autosomal dominant *FGFR2*-related phenotypes listed in OMIM.

Disease	Clinical Signs	
Crouzon syndrome OMIM 123500	Craniosynostosis, hypertelorism, exophthalmos and external strabismus, hypoplastic maxilla, and prognathism	Syndromic craniosynostosis
Apert syndrome OMIM 101200	Craniosynostosis, midface hypoplasia, and syndactyly of the hands and feet	Syndromic craniosynostosis
Pfeiffer syndrome OMIM 101600	Craniosynostosis syndrome with characteristic anomalies of the hands and feet	Syndromic craniosynostosis
Saethre–Chotzen Syndrome OMIM 101400	Craniosynostosis, facial dysmorphism, and hand and foot abnormalities. Hearing loss, limb anomalies, short stature and vertebral fusions	Syndromic craniosynostosis
Jackson–Weiss Syndrome OMIM 123150	Premature fusion of the cranial sutures as well as radiographic anomalies of the feet	Syndromic craniosynostosis
Antley–Bixler Syndrome without genital anomalies or disordered steroidogenesis OMIM 207410	Craniosynostosis, radio-humeral synostosis, midface hypoplasia, choanal stenosis or atresia, and multiple joint contractures	Syndromic craniosynostosis
Beare–Stevenson cutis gyrata syndrome OMIM 123790	Furrowed skin disorder of cutis gyrata, acanthosis nigricans, craniosynostosis, craniofacial dysmorphism, digital anomalies, umbilical and anogenital abnormalities, and early death. Cloverleaf skull can be observed	Syndromic craniosynostosis
Bent Bone Dysplasia Syndrome OMIM 614592	Poor mineralization of the calvarium, craniosynostosis, dysmorphic facial features, prenatal teeth, hypoplastic pubis and clavicles, osteopenia, and bent long bones	Lethal skeletal dysplasia, syndromic craniosynostosis
LADD syndrome 1 OMIM 149730	Affecting lacrimal glands and ducts, salivary glands and ducts, ears, teeth, and distal limb segments	Multiple congenital anomaly disorder

**Table 2:** Comparison between clinical and genetic data from this report and Dantsev et al.

	This Report (Proband)	Dantsev et al. (Proband)
**Initial clinical diagnosis**	Osteogenesis Imperfecta	Osteogenesis Imperfecta
**Age**	11 y.o.	13 y.o.
**Family history**	Negative	Positive
**Birth parameters**	Weight: 3680 g.	Weight: 2750 g.
	Length: 52 cm	Length: 55 cm
	Apgar score: 9–10	Apgar score: 8–9
**Age at first fracture**	6 y.o.	1 y.o.
**Site of first fracture**	Tibial metaphysis (left)	Hand finger (distal phalange)
**Fracture sites**	Tibial metaphysis (6 y.o.)	Hand fingers (1 and 12 y.o.)
	T3, T4, T5 vertebrae (7 y.o.)	Nose bone (3 and 4 y.o.)
	Femoral neck (7 y.o.)	Ulna and Radius (7 y.o.)
	Tibial diaphysis (7 y.o.)	Fibula (9 y.o.)
	Radius diaphysis (8 y.o.)	Feet fingers (11 and 12 y.o.)
	Femur (8 y.o.)	Metacarpal bones (13 y.o.)
**Number of fractures**	>6	>9
**DEXA Z Score**	Lumbar Z-score = −3	Spine Z-score = −2.3
	Total body Z-score = −1.6	Total body Z-score = −1.6
**Biochemical markers of bone metabolism**	PTH: 15 pg/mL (N 14–85)	PTH: 72 pg/mL (N 16–62)
	BAP: 77.6 mcg/L (N 4–21)	BAP: 48.91 mcg/L (N 48.06–120)
	25-hydroxyvitamin D: 32.1 ng/mL (N > 30)	25-hydroxyvitamin D: 14.1 ng/mL (N 14–60)
	Calcium (total): 9.6 mg/dL (N 8.5–10.5)	Calcium (total): 2.58 mg/dL (N 2.20–2.65)
	Phosphate: 5.6 mg/dL (N 3.7–5.6)	Phosphate: 1.66 mg/dL (N 1.29–2.26)
**Teeth abnormalities**	No	Severe dental caries, decreased density of dental enamel
**Extra-skeletal clinical findings**	Protruding abdomen, acessory spleen	No
**Other skeletal features**	Valgus knee, tibia varus, pronated feet, soft skin, mild hypotonia, mild joint hypermobility	Scoliosis, *genu valgum*, flezion contracture of the ankles, *pes planus*, arthralgia
**Treatment**	Neridronate, NSAIDs, magnesium, pregabalin	Zelodronic acid, Osteogenon, colecalciferol
**Treatment efficacy**	Lack of clinical benefit	Significant BMD improvement
**Molecular findings**	*FGFR2*: c.1262G>A, p.(Arg421His)—(MOS)	FGFR2: c.722dup p.(Asn241Lysfs*43)—(HE)

y.o.: years old; BAP: bone alkaline phosphatase; N: normal range; MOS: mosaicism; HE: heterozygous.

## Data Availability

The data presented in this study are available on request from the corresponding author due to privacy, legal, and ethical reasons.
